# Association Between Plasma Exosomes S100A9/C4BPA and Latent Tuberculosis Infection Treatment: Proteomic Analysis Based on a Randomized Controlled Study

**DOI:** 10.3389/fmicb.2022.934716

**Published:** 2022-07-22

**Authors:** Ying Du, Henan Xin, Xuefang Cao, Zisen Liu, Yijun He, Bin Zhang, Jiaoxia Yan, Dakuan Wang, Ling Guan, Fei Shen, Boxuan Feng, Yongpeng He, Jianmin Liu, Qi Jin, Shouguo Pan, Haoran Zhang, Lei Gao

**Affiliations:** ^1^National Health Commission of the People's Republic of China (NHC) Key Laboratory of Systems Biology of Pathogens, Center for Tuberculosis Research, Institute of Pathogen Biology, Chinese Academy of Medical Sciences and Peking Union Medical College, Beijing, China; ^2^Center for Diseases Control and Prevention of Zhongmu, Zhengzhou, China; ^3^The Sixth People's Hospital of Zhengzhou, Zhengzhou, China

**Keywords:** latent tuberculosis infection, exosome, proteomic analysis, reversion, preventive treatment

## Abstract

**Background:**

Identifying host plasma exosome proteins associated with host response to latent tuberculosis infection (LTBI) treatment might promote our understanding of tuberculosis (TB) pathogenesis and provide useful tools for implementing the precise intervention.

**Methods:**

Based on an open-label randomized controlled trial (RCT) aiming to evaluate the short-course regimens for LTBI treatment, plasma exosomes from pre- and post-LTBI treatment were retrospectively detected by label-free quantitative protein mass spectrometry and validated by a parallel reaction monitoring method for participants with changed or not changed infection testing results after LTBI treatment. Eligible participants for both screening and verification sets were randomly selected from the based-RCT in a 1:1 ratio by age and gender. Reversion was defined as a decrease in IFN-γ levels from >0.70 IU/ml prior to treatment to 0.20 IU/ml within 1 week of treatment. The predictive ability of the candidate proteins was evaluated by receiver operating characteristic (ROC) analysis.

**Results:**

Totally, two sample sets for screening (*n* = 40) and validation (*n* = 60) were included. Each of them included an equal number of subjects with persistent positive or reversed QuantiFERON-TB Gold In-Tube (QFT) results after LTBI. A total of 2,321 exosome proteins were detected and 102 differentially expressed proteins were identified to be associated with QFT reversion. Proteins with high confidence and original values intact were selected to be further verified. Totally, 9 downregulated proteins met the criteria and were validated. After verification, C4BPA and S100A9 were confirmed to be still significantly downregulated (fold change <0.67, *p* < 0.05). The respective areas under the ROC curve were 0.73 (95% CI: 0.57–0.89) and 0.69 (95% CI: 0.52–0.86) for C4BPA and S100A9, with a combined value of 0.78 (95% CI: 0.63–0.93). The positive and negative predictive values for combined markers were 70.10% (95% CI: 50.22–86.30%) and 55.63% (95% CI: 29.17–61.00%).

**Conclusion:**

Our findings suggest that downregulated C4BPA and S100A9 in plasma exosomes might be associated with a host positive response to LTBI treatment. Further studies are warranted to verify the findings and potential underlying mechanisms in varied populations with a larger sample size.

## Introduction

About one-fourth of the world's population is estimated to be infected with Mycobacterium tuberculosis (MTB), and 5–10% of them might develop active tuberculosis (TB) during their lifetime (Houben and Dodd, 2016; World Health Organization, 2020). In the absence of an effective vaccine, except for early diagnosis and standardized treatment of active TB patients, strengthening latent tuberculosis infection (LTBI) testing and treatment among individuals at high-risk of developing the active disease is a critical tool for achieving the goals of the END TB strategy. Currently, the protective effect of preventive treatment is mainly evaluated by the reduction of the incidence. However, it usually requires a long follow-up period and high cost. Immunological methods such as interferon-gamma release assays (IGRAs) and tuberculin skin tests (TSTs) were recommended for detecting MTB infections (World Health Organization, 2015), but they have the inability of predicting the development of active diseases and treatment effects (Sia and Wieland, 2011). IGRA was frequently studied to monitor the treatment efficacy of active TB (Carrara et al., 2004; Lee et al., 2010; Chiappini et al., 2012). Our previous study found QuantiFERON-TB Gold In-Tube (QFT) reversion also occurred in the untreated group, however, it was indeed related to a low risk of TB development as compared with QFT persistent positives (Xin et al., 2020). Additionally, although some studies have been engaged in exploring the potential biomarkers for reflecting LTBI treatment efficacy, very few have been well-validated and further used in practice. Therefore, it is warranted to further explore potential biomarkers to evaluate the performance of LTBI treatment.

Exosomes are 30–100 nm membrane-bound vesicles, which are constitutively released from most eukaryotic cell types into the lymphatic system and blood to facilitate systemic and local intercellular communication (Johnstone et al., 1987). Their contents were attributed to the cell of origin, reflecting cellular abnormalities and disease states (Borges et al., 2013). Previous studies suggest that exosomes might be associated with immune regulation and the pathogenic progression of MTB (Diaz et al., 2016). Another study using proteomics analysis has reported that exosomes were released from the bacillus and infected cells in TB patients, and MTB infection significantly influenced the host protein composition of circulating exosomes (Biadglegne et al., 2022). In addition, some related MTB proteins were also found to be carried by exosomes (Giri et al., 2010), and some of them were related to MTB toxicity (Lee et al., 2015). These studies suggest that if we could identify the dynamic changes and mediated pathways of the exosome proteins during TB progression, it could provide new insights into developing host-directed therapy and improving TB control. However, few studies have been conducted to explore circulating exosomes for evaluating LTBI treatment. Therefore, the aim of this pilot study was to explore and validate the differentially expressed exosome proteins associated with LTBI treatment and to explore potential markers that could be used for reflecting host response to LTBI treatment using quantitative proteomic techniques and parallel reaction monitoring (PRM).

## Materials and Methods

### Study Participants

The present study was a nested case-control study design based on an open-label randomized controlled trial (RCT) between 20 October 2018 and 30 November 2018. This RCT was initially designed to evaluate the effect of an ultra-short regimen among prior TB patients in rural Chinese residents (this RCT was registered at the Chinese Clinical Trial Registry with identifier ChiCTR-1800018224). At baseline, eligible participants were those without current active TB and with an QFT (Qiagen, United States) positive result (TB Ag-Nil ≥ 0.35 IU/ml). Active TB cases were defined according to the National Guideline for the Diagnosis of Pulmonary Tuberculosis (WS 288-2017). To ensure the included subjects were stable prior TB, repeated chest radiographs were performed at intervals of 3–6 months to exclude the participants in stages of subclinical TB or early TB. In addition, to minimize the potential influence of basic health conditions among study participants, individuals with underlying diseases such as cancer (which was not specified in this study, including all malignant tumors), liver dysfunction or impairment, renal insufficiency or degeneration, HIV infection or AIDS patients were excluded. During treatment, the intervention group received a 6 weeks regimen of twice-weekly rifapentine (RPT) plus isoniazid (INH), with a maximum dose of 600 mg for each. The control group comprised untreated controls. In the current study, eligible participants for both screening and verification sets were randomly selected from the based-RCT in a 1:1 ratio by age and gender. Eligibility for the current study required (1) completion of the assigned regimens and (2) a baseline IFN-value of 0.70 IU/ml. Reversion might be increasingly occurred in subjects with original testing results close to the diagnostic cut-off value, especially within a borderline zone between 0.2 and 0.7 IU/ml (Rafiza and Rampal, 2012). Therefore, we used a much stricter definition of reversion: the IFN-γ level of TB antigen-Nil (TB Ag-Nil) decreased from >0.70 IU/ml before the start of LTBI treatment to <0.20 IU/ml at a 1-week post-LTBI treatment.

### Exosomes and Protein Measurements

Blood samples before and after LTBI treatment have been collected and retained. The isolated exosomes were measured by nanoparticle tracking analysis (NTA) (Kreutel and Linz, 2018) using the ZetaVIEW® (Particle Metrix, Germany) equipment. The morphological characteristics were observed by transmission electron microscope (TEM, FEI, Tecnai G2 Spirit BioTwin). The identified proteins were quantified with the Bicinchoninic Acid Assay (BCA). A mass spectrometry (MS) analysis was conducted and a sequent HT search algorithm in Proteome Discoverer Software 2.4 was used for protein identification and quantification (Thermo Fisher, Waltham, MA, USA) (Eng et al., 1994). A 1.5-fold change (FC) (or 0.67) and *p* < 0.05 were adopted to identify differentially expressed proteins between groups. Only proteins that met all the following criteria were further selected as candidate proteins: First, exosome proteins showed significant differences before and after preventive treatment in the QFT reversion group. Second, there was no difference before and after treatment in the persistent positive group. Third, there was no difference between the two groups before preventive treatment. A PRM analysis was performed to validate the differentially expressed proteins. Detailed procedures are shown in Supplementary File 1.

### Statistical Analysis

Statistical analyses were performed using SAS 9.4 version (SAS Institute Inc., NC, USA) and GraphPad Prism 9 (GraphPad Software, San Diego, CA). The numerical variables were presented with a median (Q25–Q75). A Wilcoxon rank sum test was used to compare quantitative differences across groups. The Chi-square test was used to compare the distribution of categorical variables across groups.

Volcano plots and cluster analysis were conducted to explore the most significantly differential proteins. In order to analyze the bioinformatics function of differential proteins, Gene Ontology (GO) (database version: go_201504.obo) analysis with molecular function (MF), cellular component (CC) and biological process (BP) for functional annotation was used. The Kyoto Encyclopedia of Genes and Genomes (KEGG) pathway database was then mapped to explore the pathways of the proteins. Using the String database to predict the interaction of candidate proteins (http://string-db.org/, ver. 10.5).

To assess the predictive ability of the candidate proteins, a receiver operating characteristic (ROC) analysis was conducted and the area under the ROC curve (AUC) was calculated using the trapezoidal rule (Rosner, 2005). Sensitivities [probability of being test “positive” when reversion present = true reversion/(true reversion + falsely persistent positive)] and specificities [probability of being test “negative” when reversion absent = truly unreversed/(truly unreversed + false reversion)] were also calculated using the highest Youden Index (YI = sensitivity+specificity-1, also called as diagnostic accuracy index) value as the cut-off. In addition, positive predictive value [PPV, percentage of participants with a “positive” test whose QFT results were actually reversed = true reversion/(true reversion + false reversion)], negative predictive value [NPV, percentage of participants with a “negative” test whose QFT results were not reversed = truly persistent positive/(truly persistent positive + falsely persistent positive)], positive likelihood ratio [+LR = sensitivity/(1–specificity)], and negative likelihood ratio [–LR = (1–sensitivity)/specificity] were all calculated. If there were two or more eligible candidate proteins after verification, general discriminant analysis (GDA) with the Bayes posterior probability method would be used to evaluate the combined performance of single candidate proteins using SPSS software version 20.0 (SPSS, Chicago, IL). A two-tailed *p* < 0.05 was considered statistically significant.

## Results

### Characteristics of the Study Participants

**Figure 1:**
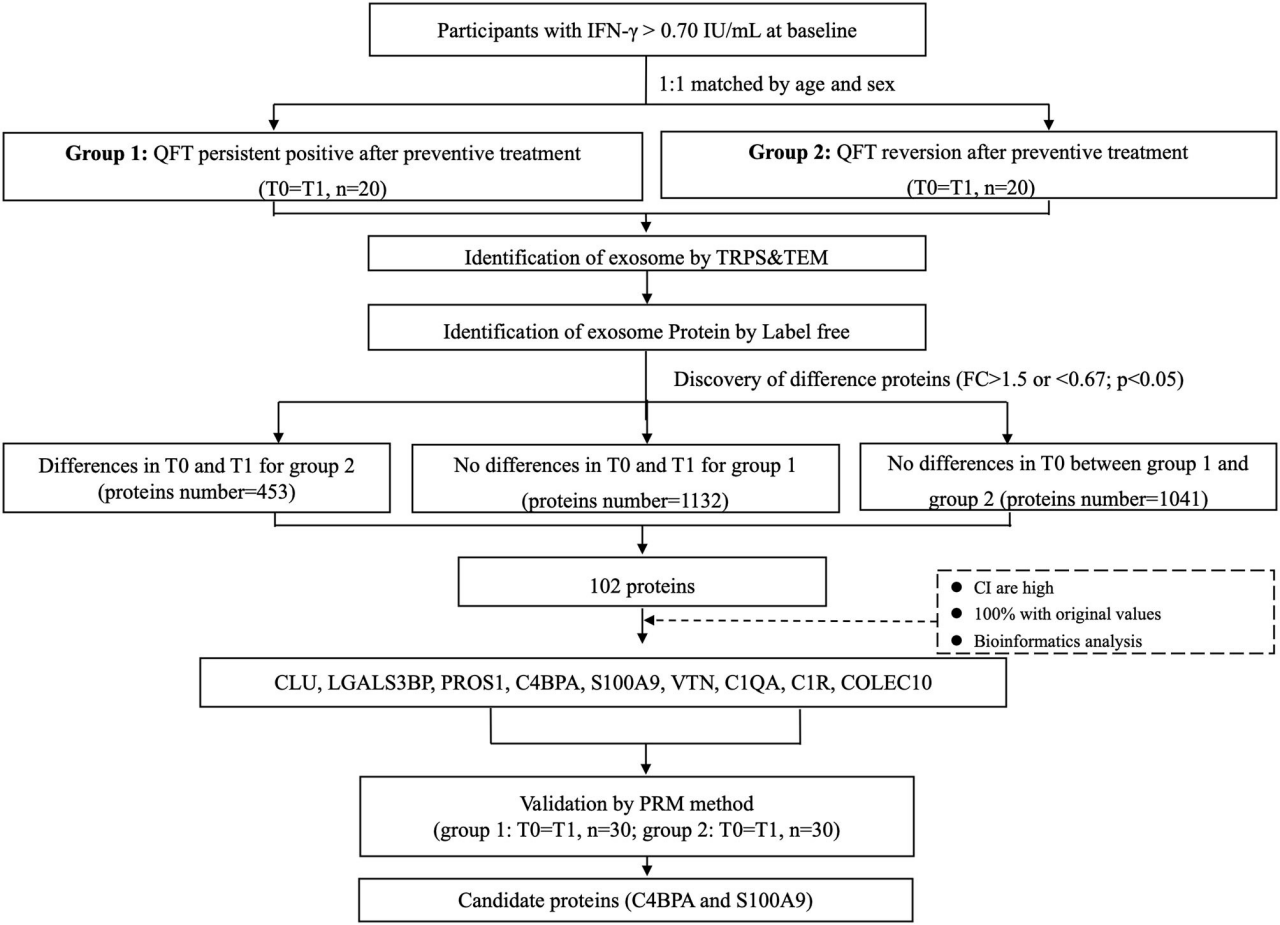
Sample selection and screening process of candidate proteins. Totally, 20 subjects with reversed QFT results after LTBI treatment and 20 age and gender-matched controls with persistent positive QFT results after LTBI treatment were included in the screening set. For each of them, circulating exosomes were collected for proteomic analysis from serums pre- (T0, before LTBI treatment) and post- (T1, after LTBI treatment) LTBI treatment respectively. A total of 102 differentially expressed proteins were identified to be associated with QFT reversion. Among them, 9 downregulated proteins were validated in the verification set (*n* = 60, with 120 samples), including CLU, LGALS3BP, PROS1, C4BPA, S100A9, VTN, C1QA, C1R, and COLEC10. Finally, C4BPA and S100A9 were confirmed to be still differentially downregulated (FC <0.67, *p* < 0.05). FC, fold change; LTBI, latent tuberculosis infection; QFT, QuantiFERON-TB Gold In-Tube.

Sample selection and screening process of proteins are shown in Figure 1 and Supplementary Figure 1. Totally, for the preliminary screening sample set, 20 subjects with reversed QFT results after LTBI treatment and 20 controls with persistent positive QFT results after LTBI treatment were included. For each of them, circulating exosomes were collected for proteomic analysis from plasma pre- and post-LTBI treatment, respectively. For the verification set, 30 QFT persistent positive and 30 reversion participants were randomly selected using the same criteria as the preliminary screening set in the same cohort. A total of 120 plasma samples collected before and after LTBI treatment, respectively, were tested with the PRM method.

Table 1 shows the major characteristics of the study participants. For the proteomic set, no significant differences were found between the QFT reversion group and the QFT persistent positive group with respect to gender, age, median body mass index (BMI) level, smoking, alcohol drinking, baseline INF-γ levels, history of type II diabetes, HBsAg positive status, history of silicosis and other pulmonary diseases. For the validation set, significant differences were found between the two groups in alcohol drinking (*p* = 0.024) and INF-γ levels after LTBI treatment (*p* < 0.001). Additionally, there were no history of organ transplantation, autoimmune diseases, or undergoing treatment with immunosuppressive agents as self-reported for the two sample sets.

**Table 1:** Characteristics of the participants included in the study.

**Variables**	**Proteomic set**			**Validation set**		
	**QFT persistent positive**	**QFT reversion**	***P***	**QFT persistent positive**	**QFT reversion**	***P***
Median age (Q25–Q75) (years)	61.5 (51.5–67.5)	62.0 (52.5–68.0)	1.000^†^	56.7 (51.7–62.3)	57.3 (51.8–61.6)	0.796
**Gender**, ***n*** **(%)**						
Male	12 (60.00)	12 (60.00)	1.000^#^	20 (66.67)	16 (53.33)	0.292
Female	8 (40.00)	8 (40.00)		10 (33.33)	14 (46.67)	
BMI (median, Q25–Q75) (Kg/m^2^)	23.50 (21.33–26.25)	24.75 (22.75–27.07)	0.344^†^	25.26 (23.53–27.16)	25.27 (23.88–27.23)	0.980^#^
**Smoking status**						
Yes	13 (65.00)	15 (75.00)	0.490^#^	10 (33.33)	11 (36.67)	0.787^#^
No	7 (35.00)	5 (25.00)		20 (66.67)	19 (63.33)	
**Alcohol drinking**						
Yes	6 (30.00)	4 (20.00)	0.465	13 (43.33)	5 (16.67)	0.024^#^
No	14 (70.00)	16 (80.00)		17 (56.67)	25 (83.33)	
**Median INF-γ** **release of QFT (Q25–Q75) (IU/ml)**						
T0	1.57 (0.89–4.76)	1.26 (0.95–2.39)	0.946^†^	1.98 (1.49–2.91)	2.08 (1.30–2.98)	0.991
T1	1.62 (0.87–4.96)	0.06 (0.00–0.13)	<0.001^†^	1.60 (1.11–3.00)	0.05 (−0.01–0.16)	<0.001^†^
*P*-Value	0.636^¶^	<0.001^†^		0.982	<0.001^†^	
History of type II diabetes*						
Yes	0 (0)	1 (5.00)	1.000^§^	1 (3.33)	2 (6.67)	1.000^§^
No	20 (100.00)	19 (95.00)		29 (96.67)	28 (93.33)	
**HBsAg positive**						
Yes	0 (0)	0 (0)	–	0 (0)	1 (3.33)	1.000^§^
No	20 (100.00)	20 (100.00)		30 (100.00)	29 (96.67)	
**History of silicosis**						
Yes	10 (50.00)	9 (45.00)	0.752^#^	9 (30.00)	14 (46.67)	0.184^#^
No	10 (50.00)	11 (55.00)		21 (70.00)	16 (53.33)	
**History of other pulmonary diseases** ^**‡**^						
Yes	0 (0)	1 (5.00)	1.000^§^	0 (0)	0 (0)	–
No	20 (100.00)	19 (95.00)		30 (100.00)	30 (100.00)	

*†Wilcoxon rank sum test*.

*^#^Chi-square test*.

*^§^Fisher exact test*.

*^¶^Comparison of baseline INF-γ release of QFT between persistent positivity and reversion*.

*History of self-report type II diabetes or fasting blood glucose ≥7 mmol·L^−1^.

*^‡^History of other pulmonary diseases referred to asthma, chronic bronchitis, chronic obstructive pulmonary disease, emphysema, and lung abscess. Autoimmune diseases in this study include hyperthyroidism, systemic lupus erythematosus, rheumatoid arthritis, ankylosing spondylitis, ulcerative colitis, systemic vasculitis, and myasthenia gravis. However, no participants with organ transplantation, autoimmune diseases or undergoing treatment with immunosuppressive agents as self-reported for two sample sets were found, therefore, related data are not shown in Table 1*.

*BMI, Body mass index; QFT, QuantiFERON-TB Gold In-Tube; Q25, Quartile 25; Q75, Quartile 75*.

### Isolation and Identification of Plasma Exosomes

As shown in Supplementary Figure 2, tunable resistive pulse sensing (TRPS) results showed the exosome diameter was concentrated. The average size of isolated exosomes was found to be 99–123 nm, which was consistent with the theoretical exosomes' diameter. TEM results showed they have a disc shape and the purity was perfect.

### MS Identification of Proteins

The distribution of the molecular weights (M*r*) of the identified proteins was depicted in Supplementary Figure 2. A total of 2,321 proteins were identified by searching the Uniprot database. The M*r* ranged from 1.4 to 3,813.7 kDa, and nearly half (1,129 of 2,321) were between 20 and 60 kDa. The proportion of the proteins with ≥ 2 unique peptides was 65.32% (1,516/2,321). The maximum peptide length was 9 and the average length was 12.59, which was in line with the reasonable range of peptide length. The protein identification was filtered to a high peptide confidence value and <1% (0.62%, 7/1,129) cumulative false discovery rate (FDR) at the peptide level.

### Identification of Candidate Proteins

**Figure 2:**
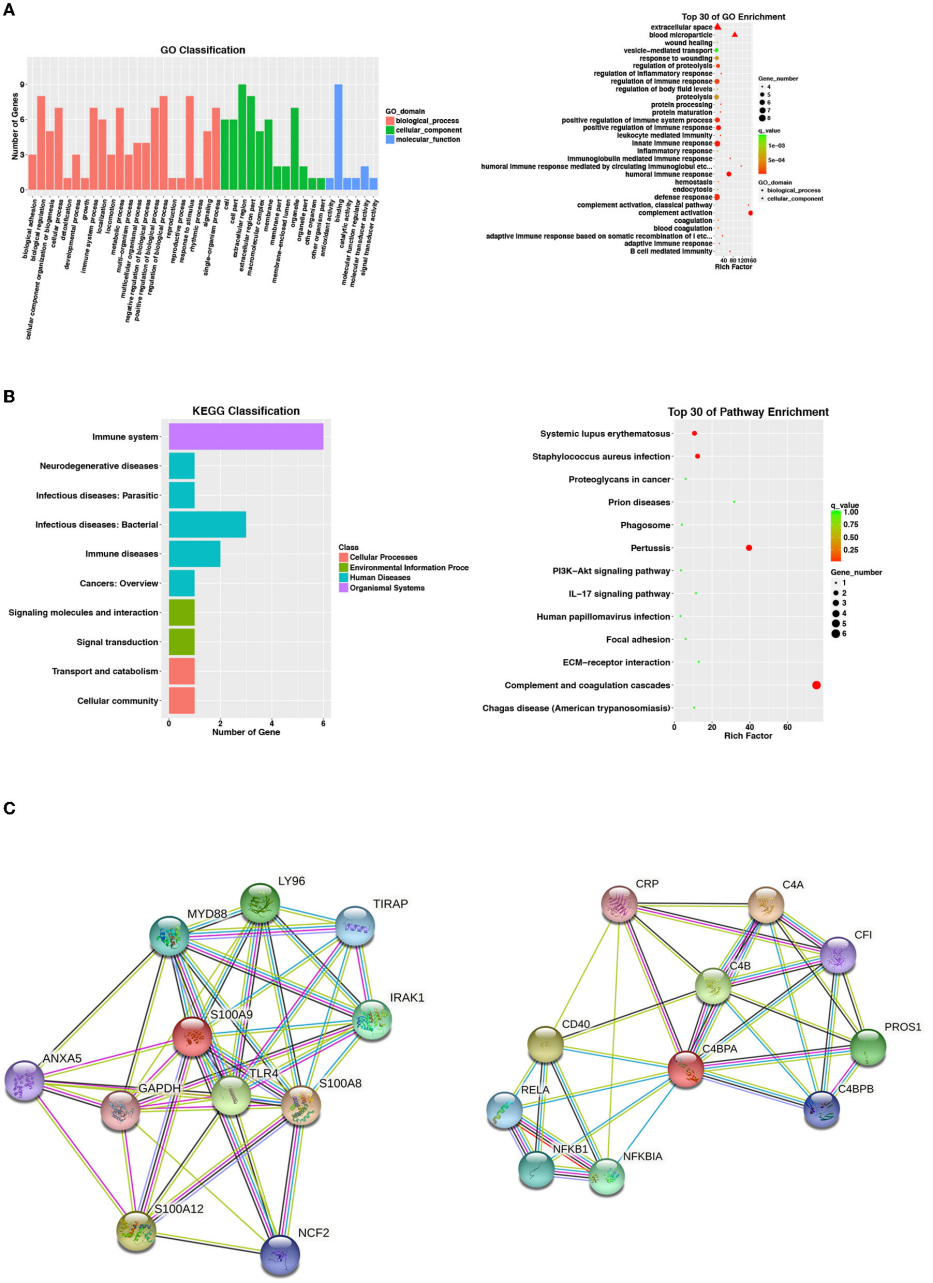
Proteomic analysis of nine identified differentially expressed proteins. For the two analyses of **(A,B)**, selecting no more than 30 items with the smallest *p*-value to draw the bar plot and dot plot, respectively. **(A)** Go enrichment analysis by biological process, cell component, and molecular function for 9 identified differentially expressed proteins. Bar chart showing the significant proteins involved in the distribution of biological process (BP), cellular component (CC), and molecular function (MF). **(B)** Kyoto Encyclopedia of Genes and Genomes (KEGG) classification and pathway analysis of 9 identified differentially expressed proteins. Bar chart representing the classification of enriched KEGG pathways. The scattered plot showed the top 30 of pathway enrichment. The X-axis indicates the rich factor; Y-axis indicates the name of the KEGG pathway. The dot size means the protein number and the dot color indicates the *p*-value. **(C)** String.org protein–protein interaction network for C4BPA and S100A9. Light blue line, known interaction from curated database, pink line, known interaction experimentally tested; dark blue line, gene co-occurrence; dark green line, predicted gene neighborhood; light green line, text mining interaction source; black line, co-expression interaction source; light purple line, protein homology interaction source. BP, biological process; CC, cellular component; GO, gene ontology; KEGG, Kyoto encyclopedia of genes and genomes; MF, molecular function.

A total of 102 differentially expressed proteins were identified to be associated with QFT reversion. Among them, we selected proteins with high confidence and original values intact to be further verified. Totally, nine downregulated proteins met the criteria and were validated in an independent sample (*n* = 60), including CLU, LGALS3BP, PROS1, C4BPA, S100A9, VTN, C1QA, C1R, and COLEC10 (Table 2). Based on the GO analysis aiming to explore their potential biological function, the results indicated that the differentially downregulated proteins were mostly enriched in innate or humoral immune response and defense response. KEGG pathway analysis suggested that they were mainly enriched in bacterial infectious diseases (Figures 2A,B).

**Table 2:** The basic information of differential expressed proteins across groups.

**No**.	**Gene symbol**	**T1 vs. T0 in group 2**		**T1 vs. T0 in group 1**		**T0 in group 1 vs. T0 in group 2**		**Accession**	**Description**	**Sum PEP score**	**Unique peptides**	**AAs**	**MW [kDa]**
		**FC**	***P***	**FC**	**P**	**FC**	***P***						
1	CLU	0.64580	0.0453	0.86204	0.4914	0.87205	0.4644	P10909	Clusterin	260.092	30	449	52.5
2	LGALS3BP	0.63639	0.0279	0.82519	0.3085	0.95133	0.7650	Q08380	Galectin-3-binding protein	181.207	22	585	65.3
3	PROS1	0.59344	0.0258	0.89155	0.6068	0.85208	0.4106	P07225	Vitamin K-dependent protein S	244.158	27	676	75.1
4	C4BPA	0.58868	0.0200	1.05610	0.7486	0.99235	0.9648	P04003	C4b-binding protein alpha chain	424.265	42	597	67
5	S100A9	0.57972	0.0265	0.76101	0.3623	0.71234	0.1256	P06702	Protein S100-A9	46.474	10	114	13.2
6	VTN	0.56940	0.0164	0.89093	0.5885	0.87429	0.4761	P04004	Vitronectin	106.167	14	478	54.3
7	C1QA	0.50114	0.0037	0.96542	0.8725	0.77577	0.1825	P02745	Complement C1q subcomponent subunit A	28.093	5	245	26
8	C1R	0.41773	0.0037	0.78246	0.3507	0.84031	0.4146	P00736	Complement C1r subcomponent	284.453	35	705	80.1
9	COLEC10	0.30430	0.0001	0.74100	0.2650	0.71780	0.0923	Q9Y6Z7	Collectin-10	25.204	6	277	30.7

*FC, Fold change; Group 1, QuantiFERON-TB Gold In-Tube (QFT) persistent positive; Group 2, QFT reversion; T0, At baseline; T1, After treatment*.

*Sum PEP Score: Displays the scores that the Protein FDR Validator node calculates on the basis of the PEP values of the PSMs. The application uses these scores to rank the list of proteins. AAs: Displays the length of the protein sequence. MW [kDa]: Displays the calculated molecular weight of the protein. The application calculates the molecular weight without considering PTMs*.

### Validation of Candidate Proteins

After PRM verification, 8 out of 9 proteins were captured (CLU, LGALS3BP, PROS1, C4BPA, S100A9, VTN, C1QA, and C1R) and PROS1 exhibited a converse regulation direction. C4BPA and S100A9 were confirmed to be differentially downregulated after LTBI treatment. The levels of these two proteins before and after LTBI treatment were shown in Supplementary Figure 3.

After searching the STRING database for predicted functional partners (string-db.org), however, no close interactions were found between C4BPA and S100A9 or with other identified differential proteins (Figure 2C).

### Predictive Performance of Candidate Proteins

**Figure 3:**
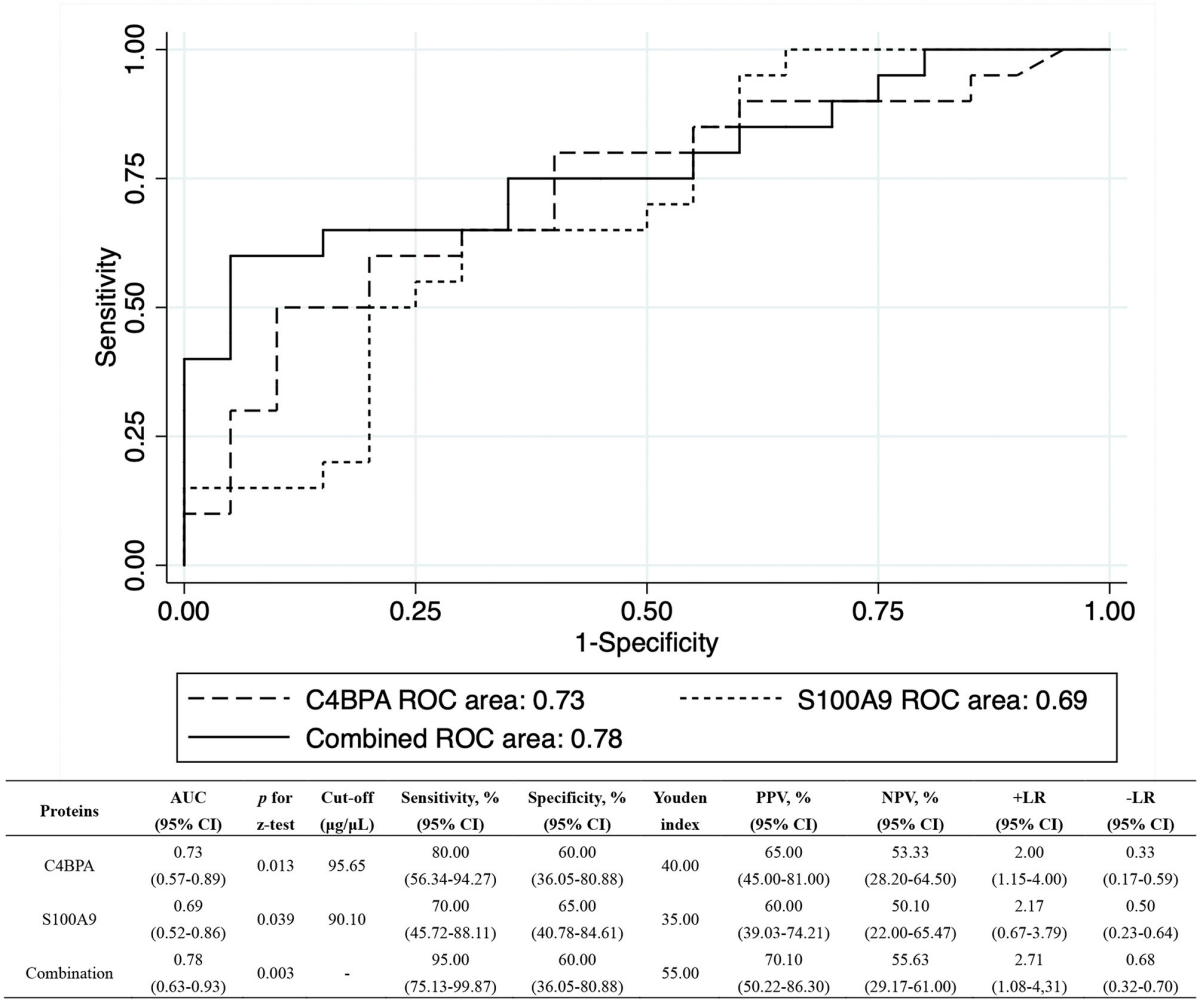
The ROC analysis for C4BPA and S100A9 using proteomic set. The ROC analyses were performed for baseline levels of C4BPA and S100A9 among the QFT reversion group, respectively. Their respective AUCs were 0.73 (95% CI: 0.57–0.89) and 0.69 (95% CI: 0.52–0.86). The combined AUC of C4BPA and S100A9 increased to 0.78 (95% CI: 0.63–0.93). AUC, area under ROC curve; CI, confidence interval; +LR, positive likelihood ratio; –LR, negative likelihood ratio; NPV, negative predictive value; PPV, positive predictive value; QFT, QuantiFERON-TB Gold In-Tube; ROC, receiver operating characteristic.

To further evaluate the predictive capability, GDA was applied to combine the C4BPA and S100A9 (Wilks' lambda: 0.376, *p* < 0.001), and ROC analyses were performed among the QFT reversion group before LTBI treatment using the preliminary screening samples. Figure 3 showed the detailed performance parameters of ROC analysis. The respective AUC values were 0.73 (95% CI: 0.57–0.89) and 0.69 (95% CI: 0.52–0.86) for C4BPA and S100A9 with a combined AUC value of 0.78 (95% CI: 0.63–0.93). The two combined biomarkers resulted in the accurately predicted 55.00% of QFT reversion with 95.00% (95% CI: 75.13–99.87%) sensitivity and 60.00% specificities (60.00%, 95% CI: 36.05–80.88%). PPV and NPV were also calculated to reflect the proportion of negative or positive results after treatment that were relatively true negatives or positives. It was found to be 70.10% (95% CI: 50.22–86.30%) and 55.63% (95% CI: 29.17–61.00%) of PPV and NPV for combination, respectively. The +LR and –LR were 2.71 (95% CI: 1.08–4.31) and 0.68 (95% CI: 0.32–0.70).

## Discussion

In this pilot study, nine downregulated proteins were found to be significantly expressed with QFT reversion after LTBI treatment. After validation, C4BPA and S100A9 were confirmed to be differentially downregulated post preventive treatment. Moderate predictive performance was observed with a combined AUC value of 0.78. It suggested that downregulated C4BPA and S100A9 in plasma exosomes might play a potential role in reflecting the host immune response to LTBI treatment.

To our knowledge, there are few studies focused on the association between plasma exosome proteins and the performance of LTBI treatment. There are some advantages to our study. The first is plasma-derived exosomes were used in this study, in which the relatively simple composition might be greatly beneficial in discovering potential markers, as compared with serum or plasma specimens. Because the interferences with high abundance of proteins in plasma and self-contained vesicles in the serum were frequent. On the other hand, a total of 2,321 proteins were identified using label-free quantitation in the current study. More importantly, we identified a list of vesicular proteins derived from the cell membrane, cell or cell part, or extracellular regions. Proteins located at these sites might contribute to the MTB pathogenesis (Bhatnagar et al., 2007; Lee et al., 2015; Mehaffy et al., 2017). Therefore, using the PRM validation method with a high resolution and high mass accuracy mode (Bourmaud et al., 2016), differentially expressed proteins in our findings may provide more evidence to explore promising biomarkers associated with the host immune response post-LTBI treatment.

Our study showed that the expression of S100A9 was differentially downregulated after preventive treatment in the QFT reversion group. But such a trend was not found among the QFT persistent positive group. The mediating role of S100A9 has been widely studied in diseases' pathogenesis. The S100A9-RAGE-NF-κB-JunB pathway in brain metastasis has been identified as a potential mediator of brain radiotherapy resistance in the organ (Monteiro et al., 2022). S100A9-CXCL12 signaling with an αPD-1 antibody could be effectively suppressed by the treatment of inhibitors for breast cancer (Li et al., 2022). S100A9 overexpression in obesity impaired macrophage differentiation *via* TLR4-NFkB signaling, worsening inflammation and wound healing (Franz et al., 2022). S100A9-ALDH1A1-RA signaling pathway that drives lethal brain relapse and could be targeted by pan-RAR antagonists to prevent cancer progression (Biswas et al., 2022). A microRNA-21-mediated SATB1/S100A9/NF-κB axis has been reported to promote chronic obstructive pulmonary disease pathogenesis (Kim et al., 2021). Of note, S100A9 was up-regulated in TB patients, which suggested its damage-associated molecular-pattern features (Kuipers et al., 2013; Scott et al., 2020). Currently, the potential role of varied S100A9 expression as a diagnostic biomarker for TB has been gradually reported (Xu et al., 2015; Kundu et al., 2020; Robak et al., 2022). The high expression of plasma S100A9 was also found to be associated with the death risk of severe TB patients (Liu et al., 2021). S100A9 could also play a key role in granuloma formation (Yoshioka et al., 2016). These studies consistently suggested that S100A9 might exert potential effects during disease progression. The mechanism of S100A9 in TB pathogenesis may operate by regulating the expression of the integrin CD11b, which is required for neutrophil accumulation in the lung (Scott et al., 2020). Conversely, the phenomenon of inflammatory disease was trending toward decreased inflammation when S100A9 deficiency, to some extent, was consistent with our results of an association between the downregulated S100A9 and QFT reversion. Our results might suggest that S100A9 can be used as a potential marker to reflect the instant host immune response to LTBI treatment through related inflammatory reactions. Nevertheless, studies with large sample sizes and related pathogenesis are needed to verify the current results.

C4BPA was another differentially downregulated protein after validation in the present study. C4BPA, a gene controlling the canonical pathway of complement activation, was usually thought to form part of the extracellular complement regulator C4b-binding protein (C4BP) (Antoniades et al., 2009). C4BPA has binding sites for many ligands, such as C-reactive protein and heparin, which are key molecules involved in inflammatory and coagulation pathways (Brodeur et al., 2003). C4BPA was also found to be a risk factor for venous thrombosis *via* an unknown protein S-independent mechanism (Buil et al., 2010). C4BP-IgM could be used as a conventional antibacterial agent to assist in the treatment of Neisseria gonorrhoeae infection (Bettoni et al., 2019). Group A streptococci could block the lethality of phagocytes by binding to C4BP and this points out the way for vaccine design (Buil et al., 2010; Buffalo et al., 2017). In our KEGG pathway analysis, “complement and coagulation cascades” were more significantly enriched. The complement system is a key player in innate immunity, which provides protection against pathogens without the need for previous exposure and/or immunization, in addition to potentiating adaptive immunity (Carroll and Sim, 2011). Additionally, MTB was known to interact with components of the innate immunity such as toll-like receptors, complement, surfactant proteins SP-A, and SP-D (Ferguson et al., 2004; Tsolaki, 2009) and could exploit complement proteins to enter into macrophages (Schlesinger et al., 1990). C4BPA could compete with complement factor H to bind MTB, which interferes with the entry of BCG into macrophages and regulates the response of pro-inflammatory cytokines (Abdul-Aziz et al., 2016). These findings provide further evidence to support the function of C4BPA in facilitating MTB interactions with macrophages during the host-pathogen interaction. Consistent with the down-regulated expression of C4BPA in complement alternative pathway activation, our findings showed C4BPA was downregulated after LTBI treatment, which might add further evidence to consider its value as a related marker for evaluating LTBI treatment performance. A more mechanistic understanding of the role of C4BPA in TB pathogenesis among different cohorts would be warranted.

The combination of candidate markers is a way to improve the efficiency of diagnosis and prognosis, especially for markers that work in different pathways. Our results showed that no close interactions were found between C4BPA and S100A9, therefore, we tentatively combined them to reflect the capability of joint prediction. Although our results suggested that combined proteins could improve the predictive ability as compared to a single protein, no optimum potential could be achieved in this study, including inadequate PPV value. It was important to appreciate predictive values that should be interpreted with caution as they were strongly influenced by disease prevalence. When used in low TB burden settings, even using a test with a high sensitivity and specificity could have poor predictive value and therefore be of limited utility. Hence, the most potential for our combined markers in this pilot study requires a further evaluation in a large-scale investigation.

The limitations of the present study should be kept in mind. First, as shown in Supplementary Figure 3, different exosome extraction methods were used for proteomic and verified sets, which led to different levels of proteins being obtained. We could not exclude the possibility that it might influence the identification of the differently expressed proteins. Second, to explore the most promising markers, much stricter criteria were used to screen differentially expressed proteins. Some relevant proteins might be excluded. Third, IGRAs are immunological tests to identify infections, but the testing results using a single cut-off value are unstable due to the influence of the host immune level (Xin et al., 2019, 2020). Although we used a more strict definition of QFT reversion to evaluate the effect of preventive treatment, misclassification of infection status could not be comprehensively excluded. Fourth, given the present study mainly aimed to identify the instant host marker to LTBI treatment for the timely evaluation and optimization of the regimens, our results based before and 1-week post-LTBI treatment might not reflect the long-term performance of C4BPA and S100A9 in host immune response to LTBI treatment. In addition, both candidate protein testing and even correlation analysis with TB incidence after a longer follow-up are of great significance, which would be the study direction to be further explored and improved in the future. Fifth, because the based RCT targeted a population with prior TB, individuals with severe diseases such as cancer were excluded, but other chronic diseases such as diabetes, which had equilibrium comparability between the two groups, were not ruled out. Therefore, our findings could not be simply extrapolated to other populations. In addition, due to the potential heterogenicity that existed in the detection of exosome proteins and our small sample size in this study, the generalization of the study results should be cautious.

In conclusion, our results indicated the expression of C4BPA and S100A9 were significantly downregulated in plasma exosomes post-LTBI treatment. It provided new clues for using circulating exosomes as biomarkers to assess the performance of LTBI treatment. However, the findings need further validation by studies with larger sample sizes in varied populations, including studies on animals or cells addressing mechanisms.

## Data Availability Statement

The original contributions presented in the study are included in the article/Supplementary Material, further inquiries can be directed to the corresponding authors.

## Ethics Statement

The studies involving human participants were reviewed and approved by the Ethics Committees of the Institute of Pathogen Biology, Chinese Academy of Medical Sciences. The patients/participants provided their written informed consent to participate in this study.

## Author Contributions

LGa designed the study. HZ, YD, and HX organized the implementation of the study. HZ, HX, YD, BF, XC, YiH, ZL, BZ, JY, DW, LGu, FS, YoH, JL, SP, and QJ did an epidemiological investigation and quality control. HZ and YD did data management and data analysis. HZ, YD, and LGa wrote the report. All authors contributed to the review and revision and have seen and approved the final version of manuscript.

## Funding

This work was supported by the National Science and Technology Major Project of China (2017ZX10201302-002), the CAMS Innovation Fund for Medical Sciences (CIFMS) (2021-I2M-1-037), and the Fundamental Research Funds for the Central Universities (3332021092). They did not involve in trial design, patient recruitment, data collection, analysis, interpretation, or any aspect pertaining to the study.

## Conflict of Interest

The authors declare that the research was conducted in the absence of any commercial or financial relationships that could be construed as a potential conflict of interest.

## Publisher's Note

All claims expressed in this article are solely those of the authors and do not necessarily represent those of their affiliated organizations, or those of the publisher, the editors and the reviewers. Any product that may be evaluated in this article, or claim that may be made by its manufacturer, is not guaranteed or endorsed by the publisher.
