# Supplementary material for: Association Between Plasma Exosomes S100A9/C4BPA and Latent Tuberculosis Infection Treatment: Proteomic Analysis Based on a Randomized Controlled Study
Source: Front Microbiol. 2022 Jul 22;13:934716. doi: 10.3389/fmicb.2022.934716 (PMC9355536; doi:10.3389/fmicb.2022.934716)
Supplement: Supplementary file 1 [file Data_Sheet_1.docx]

**Supplementary file 1**

***Isolation and identification of exosomes***

Briefly, the 200 μl of plasma was centrifuged at 3000 xg for 15 minutes, then at 10,000 xg for 30 minutes at 4 ℃ to remove cells and debris. Next, supernatant was filtered using a 0.22-µm PVDF (Millipore). The exosomes were pelleted by ultracentrifugation twice at 110,000 xg for 90 minutes at 4 °C using a Beckman ultracentrifuge. The isolated exosomes were eluted in phosphate buffer saline (PBS) and used immediately. Measurement of exosome particle number was performed by nanoparticle tracking analysis (NTA) using the ZetaVIEW® (Particle Metrix, Germany) equipment. All instruments used a 405 nm excitation laser and were pre-calibrated for concentration with a 100 nm PSL reference standard (Applied Microspheres, Netherlands). All NTA measurements were carried out using the same camera settings as well as the same tracking parameters, with values recommended for EV detection (sensitivity: 85, Shutter: 70, min Brightness: 20, min Size: 10, max. Size: 200). Videos were taken at 30 frames per second and analysed for size and concentration using the ZetaView software (Particle Metrix, Germany).

The morphological characteristics were observed by transmission electron microscope (TEM, FEI, Tecnai G2 Spirit BioTwin) at an acceleration voltage of 80 kV. Ten μl of sample was mounted onto a carbon Formvar-coated copper grid for 5 min. The fluid was allowed to adsorb on grids to form a monolayer. Grids were stained with 1% phosphotungstic acid, 44-hydrate for 1 min. After washed thoroughly with PBS, grids were dried at room temperature for 20 min and then could be observed on the computer.

***Preparation of protein samples and protein identification***

SDT buffer (2% SDS, 7M urea, 1×protease inhibitor cocktail) was added to the sample. The lysate was sonicated and then leased for 2 h on ice. After centrifuged at 13000g for 20 min, the supernatant was quantified with the Bicinchoninic Acid Assay (BCA) Protein Assay Kit (P0012, Beyotime). For each sample, 20.88 μg of proteins were reduced with 25 mM DTT for 1 h at 57 °C. Then the detergent, DTT and other low-molecular-weight components were removed using UA buffer (8 M Urea, 150 mM Tris-HCl pH 8.5) by repeated ultrafiltration (Sartorius, 30 kD). Then 100 μl iodoacetamide (100 mM IAA in UA buffer) was added to block reduced cysteine residues and the samples were incubated for 30 min in darkness. The filters were washed with 100 μl UA buffer three times and then 100 μl 25 mM NH4HCO3 buffer twice. Finally, the protein suspensions were digested with 4 μg trypsin (Promega) in 40 μl 25 mM NH4HCO3 buffer overnight at 37 °C, and the resulting peptides were collected as a filtrate. The dried polypeptide was concentrated by centrifugation, and desalted by monospin desalination column, then dried for mass spectrometry.

Mass Spectrometry (MS) analysis were conducted on a nanoElute (Bruker, Bremen, Germany) coupled to a timsTOF Pro (Bruker, Bremen, Germany) equipped with a CaptiveSpray source. Peptides were separated on a 25cm X 75μm analytical column, 1.6μm C18 beads with a packed emitter tip (IonOpticks, Australia). The column temperature was maintained at 50°C using an integrated column oven (Sonation GmbH, Germany). The column was equilibrated using 4 column volumes before loading sample in 100% buffer A (99.9% MilliQ water, 0.1% FA) (Both steps performed at 800bar). Samples were separated at 300 nl/min using a linear gradient from 2% to 25% buffer B (99.9% ACN, 0.1% FA) over 90min before ramping to 37% buffer B (10min), ramp to 80% buffer B (10min) and sustained for 10min (total separation method time 120min). The timsTOF Pro (Bruker, Bremen, Germany) was operated in PASEF mode. Mass Range 100 to 1700m/z, 1/K0 Start 0.6 V⋅s/cm2 End 1.6 V⋅s/cm2, Ramp time 100ms, Lock Duty Cycle to 100%, Capillary Voltage 1500V, Dry Gas 3 l/min, Dry Temp 180°C, PASEF settings: 10 MS/MS scans (total cycle time 1.16sec), charge range 0-5, active exclusion for 0.4 min, Scheduling Target intensity 20000, Intensity threshold 2500, CID collision energy 42eV.

Sequent HT search algorithm in Proteome Discoverer Software 2.4 was used for protein identification and quantification (Thermo Fisher, Waltham, MA, USA). Each MS/MS spectrum was searched in human UniProt protein database (https://www.uniprot.org/proteomes/UP000005640). A mass tolerance of 10 ppm for the precursors and 0.1 Da for fragmented ions, less than two missed cleavages for trypsin specificity. Carbamidomethyl (C) was set as fixed modifications. Oxidation (M), Acetyl (Protein N-term), Met-loss+Acetyl (M), Met-loss (M) were set as variable modifications. The target false discovery rate (FDR) was calculated at 1% (strict) and 5% (relaxed), respectively. Protein abundance was calculated based on the normalized spectral protein intensity (LFQ intensity). A 1.5-fold change (FC) (or 0.667) and p < 0.05 were adopted to identify differentially expressed proteins between groups.

***Validation of differentially expressed proteins by PRM***

During verification, the equal amount peptides from each sample were mixed as quality control (QC) sample for PRM method construction. PRM analysis was performed using 3 combined fractions from QC. One µg of each fraction peptide was analyzed with the “label-free” method using the EASY-nLC^TM^ 1200 UHPLC system and Q Exactive HF-X mass spectrometer (Thermo Fisher Scientific) for 60 min. The offline raw data were searched by Proteome Discoverer v.2.2 software. The “missed cleavage” was set as 0, and 1-3 unique peptides were selected for each protein.

The selected peptides were analyzed by a “full scan” followed by a PRM pattern. The PRM was set to a resolution of 30,000 (at 200 m/z) with an AGC target value of 5×104, a maximum ion injection time of 80 ms, and an NCE of 27%. The off-line data were analyzed by Skyline software to determine, whether the selected peptides were usable based on reproducibility and stability. Equal amounts of the trypsin-treated peptide of each sample were spiked with an equal amount of the labeled peptide, DSPSAPVNVTVR, as an internal standard. Samples were analyzed by full scan followed by the PRM pattern as described above. The off-line data were analyzed by Skyline software. The peak area was corrected using the internal standard peptide.
