# Supplementary material for: Association Between Plasma Exosomes S100A9/C4BPA and Latent Tuberculosis Infection Treatment: Proteomic Analysis Based on a Randomized Controlled Study
Source: Front Microbiol. 2022 Jul 22;13:934716. doi: 10.3389/fmicb.2022.934716 (PMC9355536; doi:10.3389/fmicb.2022.934716)
Supplement: Supplementary file 2 [file Data_Sheet_2.docx]

A) Significant difference analysis（Volcano plot）


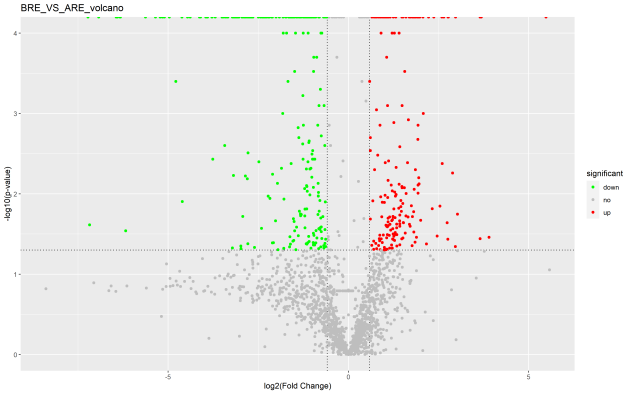

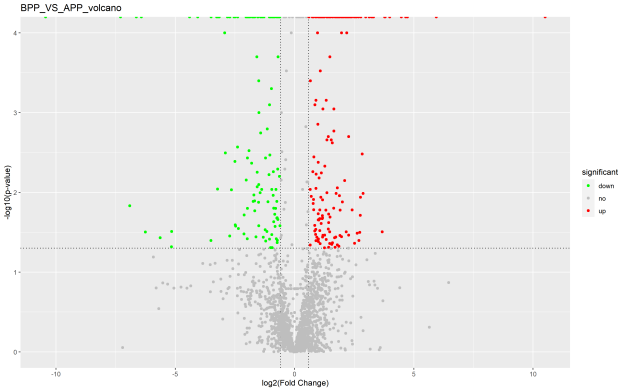


189

173

124

264

173

B) Hierarchical cluster analysis


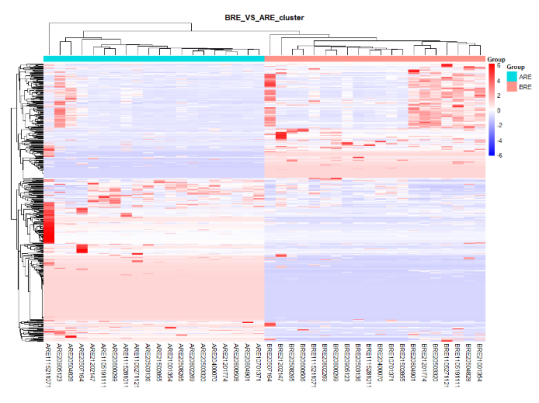

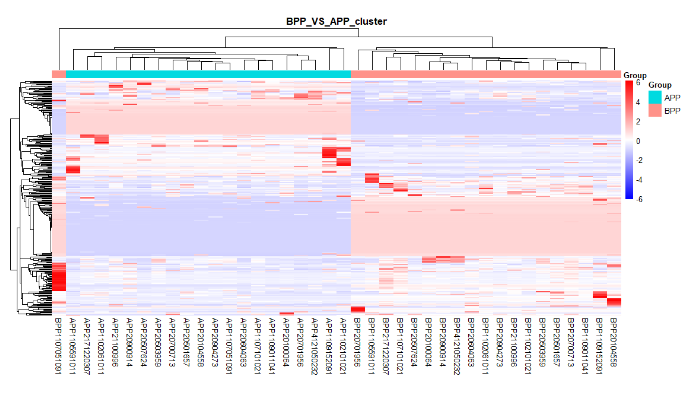


**Supplementary Figure 1 Identification and selection of plasma exosomes and proteins based on biostatistical analyses**. (A) Volcano plot was plotted by FC and p-value, in order to explore the differences between groups and help to identify the differentially expressed proteins. The -log_10_ (p-value) value indicating the vertical axis, and the log_2_ (FC) value indicating the horizontal axis. The up-regulated differential proteins are represented by red dots, down-regulated differential proteins are represented by green dots, and other proteins are represented by red and black dots. (B) The vertical axis represents the sample names between groups, and the horizontal represents the differential proteins. Red shows the high expression value of differential proteins in the clustered samples, while blue shows the low expression value of differential proteins in the clustered samples. FC = fold change.

A)







B)

C)

D)


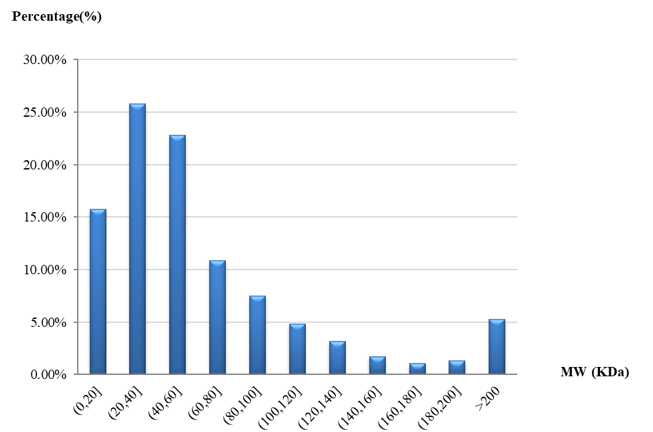

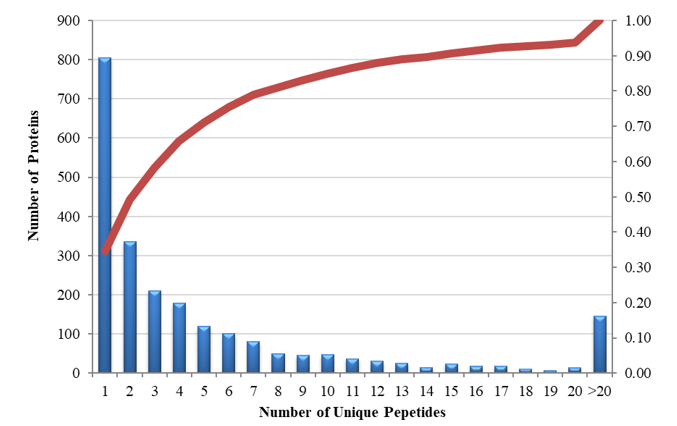

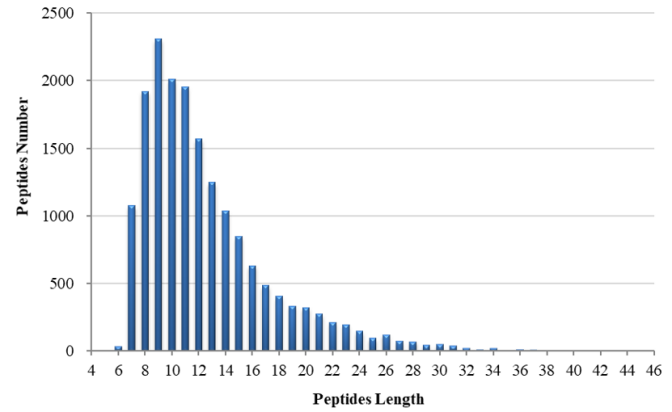


E)

| **Items** | **Num.** |
| --- | --- |
| Proteins | 2321 |
| Peptides | 17588 |
| Unique peptides | 15573 |
| Total number of profile | 313276 |

**Supplementary Figure 2 Isolation and identification of plasma exosomes and proteins**. (A) Morphology of exosomes by TEM. (B)Nanoparticle tracking detection by TRPS. (C)Standard curve of protein concentration determined by BCA method (μg /mL). (D) Distribution of molecular weight and peptides. (D) Distribution of molecular weight and peptides. (E) Results of quantitative protein expression profile. BCA = bicinchoninic acid assay. TEM = transmission electron microscopy. TRPS = tunable resistive pulse sensing.

A) B)

C) D)

**Supplementary Figure 3 The levels of C4BPA and S100A9 for proteomic set and PRM validation set (μg/μL).** A）Comparison of plasma C4BPA levels derived exosomes in pre- and post-treatment for proteomic set. The baseline C4BPA levels in T1 of reversed QFT results were significantly lower than that in T0 of persistent positive QFT results (p =0.013, Mann–Whitney U-test). However, this trend was not found when comparing between T0 and T1 group 1 (p=0.640, Mann–Whitney U-test). B）Comparison of plasma S100A9 levels derived exosomes in pre- and post-treatment for proteomic set. The S100A9 levels in T1 of group 2 were significantly lower than that in T0 of group 2 (p =0.040, Mann–Whitney U-test). However, its level was not significantly different when comparing between T0 and T1 group 1 (p=0.142, Mann–Whitney U-test). C) Comparison of plasma C4BPA levels derived exosomes in pre- and post-treatment for PRM validation set. The baseline C4BPA levels in T1 of group 2 were significantly lower than that in T0 of group 2 (p =0.001, Mann–Whitney U-test). However, this trend was not found when comparing between T0 and T1 group 1 (p=0.130, Mann–Whitney U-test). D) Comparison of plasma S100A9 levels derived exosomes in pre- and post-treatment for PRM validation set. The S100A9 levels in T1 of group 2 were significantly lower than that in T0 of group 2 (p =0.002, Mann–Whitney U-test). However, its level was not significantly different when comparing between T0 and T1 group 1 (p=0.056, Mann–Whitney U-test). Group 1 = participants with persistent positive QFT results after treatment. Group 2 = participants with reversed QFT results after treatment. PRM = Parallel Reaction Monitoring. QFT = QuantiFERON-TB Gold In-Tube. T0 = pre-treatment. T1 = 1-week post-treatment. LTBI = latent tuberculosis infection.
